# Use of a Fully Automated Internet-Based Cognitive Behavior Therapy Intervention in a Community Population of Adults With Depression Symptoms: Randomized Controlled Trial

**DOI:** 10.2196/14754

**Published:** 2019-11-18

**Authors:** Mark B Schure, Janet C Lindow, John H Greist, Paul A Nakonezny, Sandra J Bailey, William L Bryan, Matthew J Byerly

**Affiliations:** 1 Department of Health & Human Development Montana State University Bozeman, MT United States; 2 Center for Mental Health Research and Recovery Montana State University Bozeman, MT United States; 3 Department of Psychiatry College of Medicine University of Arizona Tucson, AZ United States; 4 Biomedical Research and Education Foundation of Southern Arizona Tucson, AZ United States; 5 Southern Arizona VA Health Care System Tucson, AZ United States; 6 School of Medicine and Public Health University of Wisconsin Madison, WI United States; 7 Healthcare Technology Systems Madison, WI United States; 8 Department of Cell Biology and Neuroscience Montana State University Bozeman, MT United States; 9 Department of Population and Data Science University of Texas Southwestern Medical Center Dallas, TX United States; 10 Department of Psychiatry University of Texas Southwestern Medical Center Dallas, TX United States; 11 Montana State University Extension Bozeman, MT United States; 12 One Montana Bozeman, MT United States

**Keywords:** internet-based cognitive behavior therapy, iCBT, depression symptoms, rural populations, RCT, randomized controlled trial, CBT

## Abstract

**Background:**

Although internet-based cognitive behavior therapy (iCBT) interventions can reduce depression symptoms, large differences in their effectiveness exist.

**Objective:**

The aim of this study was to evaluate the effectiveness of an iCBT intervention called Thrive, which was designed to enhance engagement when delivered as a fully automated, stand-alone intervention to a rural community population of adults with depression symptoms.

**Methods:**

Using no diagnostic or treatment exclusions, 343 adults with depression symptoms were recruited from communities using an open-access website and randomized 1:1 to the Thrive intervention group or the control group. Using self-reports, participants were evaluated at baseline and 4 and 8 weeks for the primary outcome of depression symptom severity and secondary outcome measures of anxiety symptoms, work and social adjustment, psychological resilience, and suicidal ideation.

**Results:**

Over the 8-week follow-up period, the intervention group (n=181) had significantly lower depression symptom severity than the control group (n=162; *P*<.001), with a moderate treatment effect size (*d*=0.63). Moderate to near-moderate effect sizes favoring the intervention group were observed for anxiety symptoms (*P*<.001; *d*=0.47), work/social functioning (*P*<.001; *d*=0.39), and resilience (*P*<.001; *d*=0.55). Although not significant, the intervention group was 45% less likely than the control group to experience increased suicidal ideation (odds ratio 0.55).

**Conclusions:**

These findings suggest that the Thrive intervention was effective in reducing depression and anxiety symptom severity and improving functioning and resilience among a mostly rural community population of US adults. The effect sizes associated with Thrive were generally larger than those of other iCBT interventions delivered as a fully automated, stand-alone intervention.

**Trial Registration:**

ClinicalTrials.gov NCT03244878; https://clinicaltrials.gov/ct2/show/NCT03244878

## Introduction

Depression is the leading cause of disability globally [1] and is associated with impaired function [2], higher morbidity and mortality [3,4], greater health care use [5], and higher risk of suicide [6]. In the United States, the 12-month and lifetime prevalence of major depressive disorder is 10.4% and 20.6%, respectively [2].

Cognitive behavior therapy (CBT), when delivered by clinicians, is an evidence-based psychotherapy for treating acute depression symptoms that also reduces relapse risk [7-9]. However, many barriers to receiving CBT exist, including geographic location, cost of insurance and care, long waitlists, and stigma for seeking treatment for mental illness [10]. For the approximately 46 million Americans living in rural regions [11], access to adequate mental health care is often limited or nonexistent [12]. In Montana (the focus of this study), 65.0% of residents live in rural regions [13]; the state also has a higher prevalence of depression than the national average and has had one of the highest suicide rates in the United States over the past four decades [14,15]. Despite the state’s clear need for mental health services, 60% of the population live in mental health care professional shortage areas (defined as <1 psychiatrist/30,000) and only 13.3% of the total mental health provider need is met [12].

An attractive option for treating mental disorders in rural regions is the use of affordable, internet-delivered psychotherapy that requires little or no human intervention. Several studies of traditional face-to-face CBT and internet-based CBT (iCBT), herein defined as a class of online software apps that emulate in-person psychotherapy, have shown similar effectiveness for treating depression symptoms [16-18]. Additionally, a recent meta-analysis showed comparable effectiveness of iCBT interventions for reducing depression and anxiety symptoms among urban and rural populations outside the United States [19]. These results, combined with increasing broadband access in US rural communities [20], suggest that effective iCBT interventions have the potential for widespread public health impact by expanding the availability of low-cost, effective depression treatments [21,22] and providing an attractive alternative or complementary delivery strategy for populations that face the aforementioned barriers. However, no randomized controlled trials (RCTs) of iCBT interventions for depression have been conducted among US adult rural residents [21,22].

Although, in theory, the use of iCBT interventions to treat depression symptoms in US regions lacking sufficient mental health care services is an attractive treatment strategy, determining which iCBT intervention might be most effective is complicated by the considerable differences that exist in iCBT program design (eg, static text and pictures vs video-centric formats), support (fully automated to extensive human supports), adherence, and demonstrated effectiveness for treating depression symptoms in adults with a range of symptom severities (mild to severe) and diagnoses (none, major depressive disorder, unipolar affective disorder, or dysthymia) [10,21,22]. Additionally, participant engagement has been a significant challenge for some depression iCBT interventions. Fully automated, stand-alone iCBT interventions generally have higher treatment dropout rates (74%) than those provided with therapist or administrative support (28% and 38%, respectively) [23]. Lower engagement likely decreases the effectiveness of iCBT interventions for depression. For example, in three studies that reported participants completing fewer than three mean intervention sessions, two found no significant difference in depression symptoms for the intervention compared to the control [24,25] and the third found a small effect size (0.26) [26] for depression symptoms. To date, six of the seven studies of fully automated, stand-alone versions of iCBT have demonstrated small-to-medium clinical effects [26-31] and one found no significant effects [25]. Thus, improvements in iCBT interventions and delivery strategies are needed.

The intervention evaluated in this study, Thrive, is a fully automated, stand-alone iCBT intervention designed to reduce depression symptoms using a video-based platform. The intervention incorporates classic cognitive behavior therapy themes in modules on Constructive Thinking (Cognitive Restructuring) [32], Pleasant Activities (Behavioral Activation) [33,34], and Assertive Communication (Social Skills Training) [35]. Each module consists of 10 lessons, and there is an introduction lesson that offers suggestions for choice of a first module (31 total lessons). Within each module, Thrive’s algorithms personalize content, exercises, and recommendations for participants based on their input and progress. A pilot feasibility study of Thrive in a US primary health care setting included 37 patients with depression (Symptom Checklist-20 score>1.75), of whom 59% (22/37) had suicidal thoughts at baseline (Symptom Checklist-20 item 13) [36]. At the 4-month follow-up, 52% (16/31) had ≥50% reduction in depression, 46% (14/31) had a clinically significant decrease in depression symptoms, and fewer reported having recent suicidal thoughts (35% vs 59% at baseline) [36]. Thrive was also offered to employees of four businesses located in four states. A total of 227 individuals with baseline depression symptom severity (Patient Health Questionnaire-9 item [PHQ-9] [37]) scores between 5 and 27 (mean score 10.5) and at least one follow-up PHQ-9 score logged into Thrive an average of 10.5 times and experienced an average PHQ-9 improvement of 4.4 points (42%) over an average of 7 weeks (B. Coleman, personal communication, 3 Sept 2019). Thus, preliminary data suggested that Thrive might be efficacious. Given the lack of evidence of iCBT depression interventions in rural US settings, the primary objective of this RCT was to evaluate the short-term effectiveness of Thrive to reduce depression symptom severity when delivered directly to a community population of adults with depression symptoms living in Montana, one of the least densely populated states in the United States [38]. Because the Thrive intervention required no clinician interaction or support for delivery and assessments, findings are likely predictive of the use and impact of the Thrive intervention in real-world settings and support the promise of iCBT to address unmet mental health care needs in rural US regions and possibly beyond.

## Methods

### Trial Design

An RCT compared the efficacy of the fully automated, stand-alone Thrive intervention to a waitlist control (WLC) in reducing depression symptoms among adults. Participants were recruited from communities across Montana and immediately randomized 1:1 to the intervention group (Thrive) or a WLC group (delayed access to Thrive until the 8-week follow-up assessment) after meeting the inclusion criteria and providing electronic informed consent on the study website [39] (Multimedia Appendix 1). Participants in both groups were assessed for primary and secondary outcomes at baseline and 4 and 8 weeks postenrollment. Enrollment occurred between September 2017 and January 2018, and all assessments were completed by March 2018. The Montana State University Institutional Review Board approved the protocol and all related materials (#MS033017-FC) prior to study initiation. The study is registered at ClinicalTrials.gov (NCT03244878).

### Participants

Participants were recruited using community fliers, public service announcements, local newspaper advertisements, newsletters, and community social media sites. The study was also promoted through select state organization email listservs, large employers, local health care providers, Facebook, a Craigslist community page, community meeting events, and Montana State University Extension faculty communications with their respective counties. All methods directed potential participants to a study website, which informed potential applicants about the study expectations; determined their eligibility; and guided those eligible through the informed consent, randomization, and assessment process [39].

Inclusion criteria included adults aged ≥18 years with mild-to-severe depression severity (PHQ-9 score >5) [37]; Montana residency; a valid email address; and regular access to broadband internet via a computer, tablet, or smartphone. At enrollment, potential participants who indicated recent suicidal ideation (PHQ-9 item 9 score >0) were asked to confirm that they could stay safe and those responding that they could not were considered ineligible (see the safety protocol description below for the handling of cases of suicidal ideation and Multimedia Appendix 4). All participants provided electronic informed consent prior to study participation. All participants were informed that they were free to obtain and use any additional care available throughout their participation in the trial. In total, 463 participants were enrolled, and of these, 109 were deemed fraudulent identities and their data were removed prior to analyses (Multimedia Appendix 2). Additionally, three individuals provided invalid email addresses, which led to their exclusion from study participation, and data from two control participants were discarded, as they were accidentally provided access to the intervention immediately. Six participants were excluded because of missing baseline data on the covariate “currently receiving psychosocial therapy for depression,” which was required for data analysis. The final analytic sample included 343 participants (intervention: n=181; control: n=162), of which 86 (25%) participants did not complete any follow-up assessments (Multimedia Appendices 2 and 3).

#### Intervention Group

Thrive is a fully automated, stand-alone, individually tailored iCBT intervention for depression developed by Waypoint Health Innovations [40]. Participants accessed the intervention with a Web browser or mobile app. Intervention content is largely delivered by video with minimal text and employs three structured interactive modules focused on behavioral activation, cognitive restructuring, and social skills training CBT techniques. Based on user input and usage patterns, the intervention uses algorithms to personalize feedback and tailor user progression through each therapeutic modality. Based on the study results from a qualitative study on the acceptability of Thrive [41], 6 of the 320 videos were replaced with videos depicting scenarios and settings characteristic of rural Montana to enhance engagement among the study population.

#### Control Group

The control group received a link to general depression information at the National of Institute of Mental Health [42]. Participants in the control group were also provided a link to Montana’s National Alliance on Mental Illness resource page [43]. The control group was granted access to Thrive after completing the 8-week assessment.

### Assessments

Participants completed study assessments on the study website at baseline, 4 weeks, and 8 weeks. Automated email reminders were sent when each assessment was due, with two additional follow-up automated reminder emails sent within 7 days to those who had not completed the assessment. If the participant did not complete the scheduled assessment within a 10-day window, data were considered lost to follow-up for that assessment (Multimedia Appendix 10). A US $25 Amazon gift code was sent following completion of each assessment.

### Outcomes

The primary outcome measure was participants’ self-reported depression symptom severity PHQ-9 score. Prespecified secondary outcomes included anxiety symptom severity, daily functioning, and resilience. Suicidal ideation was added as a secondary outcome after the original protocol was developed, but prior to the start of analyses.

### Measures

All outcomes were assessed using self-reported, validated measures automatically administered via the study website. Depression symptom severity was evaluated using the PHQ-9, which incorporates the major depressive disorder “A criterion” symptoms of Diagnostic and Statistical Manual of Mental Disorders (DSM)-IV and DSM-5 (score range: 0-27, with higher scores indicating worse symptoms) [37]. Anxiety symptom severity was measured with the Generalized Anxiety Disorder Scale (GAD-7, score range: 0-21), with higher scores indicating worse symptoms [44]. Daily functioning was measured using the Work and Social Adjustment Scale (WSAS, score range: 0-40), with higher scores indicating a greater impact of depression on daily functioning [45]. The abbreviated version of the Conner-Davidson Resilience Scale (CD-RISC-10) measured resilience (score range: 0-40), with higher scores indicating greater resilience [46]. Lastly, suicidal ideation was assessed using item 9 of the PHQ-9 measure (“Thoughts that you would be better off dead or of hurting yourself” in the past two weeks). Item 9 was treated as an ordinal scale that ranged from 0 (“not at all,” no suicidal ideation) to 3 (“nearly every day”) [37].

### Safety

When study applicants endorsed having at least some suicidal ideation (PHQ-9 item 9 score>0) at study enrollment, the study website displayed multiple sources of immediate help (Multimedia Appendix 4) and asked applicants to declare whether they were sure they could stay safe. Applicants answering that question negatively were considered ineligible to participate in the study. Notably, no applicants reported that they could not stay safe. Regardless of the response to the “stay safe” question, the study website prompted the individual to seek help and provided the same sources of help described in Multimedia Appendix 4. If an enrolled participant indicated s/he had at least some suicidal ideation during the 4- or 8-week assessments (PHQ-9 item 9 score >0), the study website immediately prompted the individual to seek help and provided the same sources of help. Additionally, the Thrive intervention directed individuals with PHQ-9 scores ≥20 at any assessment and those with PHQ-9 scores ≥10 on the third assessment to seek help from a doctor. A thorough description of all safety measured used is described in Multimedia Appendix 4. Participants were provided email addresses (lead investigator and IRB Chair) for reporting of any adverse experiences or events.

### Covariates

An initial pool of 10 variables was selected *a priori* for analysis as potential covariates of depression severity, anxiety, functional impairment, resilience, and suicidal ideation (Multimedia Appendix 5).

### Statistical Analysis

Outcomes were assessed at baseline and at 4 and 8 weeks. The change over time in each continuous outcome and suicidal ideation was compared between the intervention and control groups using a linear mixed model analysis of repeated measures and an ordinal logistic regression model within a Generalized Estimating Equation framework, respectively. A separate model was conducted on each outcome measure. Each model contained fixed-effects terms for treatment (intervention vs control), time, treatment × time interaction, and respective baseline measure (prior to the intervention) as covariates. Receiving therapy for depression (yes/no) at the baseline assessment was also included as a covariate in each model. Least squares means (adjusted treatment means) and adjusted odds ratios (OR) were estimated as part of the mixed model and ordinal logistic model, respectively, to interpret the treatment effect. For the ordinal logistic regression, the cumulative probabilities were modeled over the higher-ordered suicidal ideation scale scores (more suicidal ideation).

Statistical analyses were performed using SAS software, version 9.4 (SAS Institute, Inc, Cary, North Carolina). Maximum likelihood estimators allow efficient parameter estimation using only available data under an assumption of missing at random [47-49]. The level of significance was set at α=0.05 (two-tailed), and the Bonferroni method was implemented to control false-positives over the multiple tests. The 95% CIs for the point estimates of treatment group effects were also adjusted to match the Bonferroni adjustment to significance levels in the corresponding test. A priori evaluable sample size for a statistical power of 80% was estimated (Multimedia Appendix 6).

## Results

### Participant Characteristics

Participant baseline characteristics are described in Table 1. The cohort was predominately female (290/343, 85%) and Caucasian (319/343, 93%), with a mean age of 42.9 (SD 13.3) years. Most participants were married (198/343, 58%); employed, or in school (265/343, 77%); had some higher education (318/343, 93%); and lived in rural regions (287/343, 84%). In addition, 59% (202/343) of participants were receiving medication, psychosocial therapy, or other treatment for depression or anxiety at baseline. No significant differences for any outcome measure were observed at baseline (Table 2) nor did the baseline depression symptom severity differ between groups (Multimedia Appendix 7). Participants randomized to the intervention group completed a mean of 8.7 lessons. No adverse experiences or adverse events were reported by either study group.

**Table 1:** Baseline characteristics in the intent-to-treat population.

Characteristics		Total sample (N=343), n (%)	iCBT^a^ group^b^ (n=181), n (%)	Control group (n=162), n (%)
Age, mean (SD)		42.9 (SD 13.3)	42.1 (12.8)	43.8 (13.8)
Female		290 (85.0)	160 (88.4)	130 (81.2)
**Race**				
	White	319 (93.0)	170 (93.9)	149 (92.0)
	Other	24 (7.0)	11 (6.1)	13 (8.0)
**Marital status**				
	Single	145 (42.3)	78 (43.1)	67 (41.4)
	Married/domestic partnership	198 (57.7)	103 (56.9)	95 (58.6)
**Employment status**				
	Employed or student	265 (77.3)	136 (75.1)	129 (79.6)
	Retired or unemployed	78 (22.7)	45 (24.9)	33 (20.4)
Veteran		15 (4.4)	7 (3.9)	8 (4.9)
**Education**				
	High school degree or less	25 (7.3)	10 (5.5)	15 (9.3)
	Some college, bachelor or associate degree, or trade school	236 (68.8)	129 (71.3)	107 (66.0)
	Graduate or professional degree	82 (23.9)	42 (23.2)	40 (24.7)
**Health insurance**				
	Private	244 (71.1)	140 (77.3)	104 (64.2)
	Public	74 (21.6)	32 (17.7)	42 (25.9)
	Other	9 (2.6)	5 (2.8)	4 (2.5)
	None	16 (4.7)	4 (2.2)	12 (7.4)
**Rural classification^c^**				
	Urban	56 (16.3)	27 (14.9)	29 (17.9)
	Large rural	73 (21.3)	42 (23.2)	31 (19.1)
	Small rural	116 (33.8)	60 (33.2)	56 (34.6)
	Isolated	98 (28.6)	52 (28.7)	46 (28.4)
**Receiving mental health treatment^d^**				
	Yes	202 (58.9)	103 (56.9)	99 (61.1)
	No	141 (41.1)	78 (43.1)	63 (38.9)

^a^iCBT: internet-based cognitive behavior therapy.

^b^Thrive intervention group.

^c^Defined using rural urban commuting area codes [50].

^d^Defined as receiving any clinical care or taking medication(s) for depression symptoms.

**Table 2:** Baseline clinical measures for total sample and treatment groups.

Measure		Total sample (N=343), mean (SD)	iCBT^a^ group^b^ (n=181), mean (SD)	Control group (n=162), mean (SD)
**Primary outcome measure**				
	Depression symptom severity^c^	13.6 (5.0)	13.7 (5.0)	13.4 (5.0)
**Secondary outcome measures**				
	Anxiety symptom severity^d^	10.3 (4.7)	10.3 (4.7)	10.2 (4.6)
	Work and social functioning^e^	20.2 (7.9)	20.2 (8.0)	20.2 (7.8)
	Resilience^f^	22.2 (6.2)	22.2 (6.6)	22.2 (5.9)

^a^iCBT: internet-based cognitive behavior therapy.

^b^Thrive intervention group.

^c^Patient Health Questionnaire-9 score range=0-27.

^d^Generalized Anxiety Disorder Scale 7-Item score range=0-21.

^e^Work and Social Adjustment Scale score range=0-40.

^f^Connor-Davidson Resilience Scale 10-Item score range=0-40.

### Clinical Outcomes

#### Primary Outcome

Significant main effects of treatment (*F*_1,248_=28.67; raw *P*<.001, adjusted *P*<.001) and time (*F*_1,216_=11.94; raw *P*<.001, adjusted *P*<.001), favoring the Thrive intervention, were observed for depression symptom severity (Table 3), and a moderate treatment effect size (Cohen *d*=0.63, *P*<.001; Table 4) was found. The pattern of the overall least squares treatment group means showed that depression severity (following 8 weeks of intervention) was significantly lower for the intervention group than for the control group (7.702 [SE 0.336] vs 10.224 [SE 0.328], raw *P*<.001, adjusted *P*<.001; *d*=0.63; Table 4). The same pattern was observed with simple treatment group effects at weeks 4 and 8 (raw *P*<.001, adjusted *P*<.001; PHQ-9, Table 4).

**Table 3:** Main effects of treatment, time, and treatment by time interaction effects from the mixed model and ordinal logistic regression analysis for depression symptom severity, anxiety symptom severity, work/social functioning, resilience, and suicidal ideation.

Covariates and effects		Depression symptoms (PHQ-9^a^)				Anxiety symptoms (GAD-7^b^)				Functioning (WSAS^c^)					Resilience (CD-RISC-10^d^)					Suicidal ideation (PHQ-9)				
		*F* statistic (*df*)		*P* value		*F* statistic (*df*)		*P* value			*F* statistic (*df*)		*P* value			*F* statistic (*df*)		*P* value			χ^2^ (*df*)		*P* value	
Baseline outcome^e^		F (1, 245) =119.76		<.001		F (1, 253.6) =133.56		<.001			F (1, 251.7) =181.17		<.001			F (1, 247.7) =323.87		<.001			67.18 (1)		<.001	
Therapy^f^		F (1, 249) =7.58		.006		F (1, 254.1) =8.29		.004			F (1, 250.7) =8.82		.003			F (1, 243.7) =4.17		.04			3.71 (1)		.054	
**Effects**																								
	Treatment^g^		F (1, 248) =28.67		<.001; <.001^h^		F (1, 252.8) =16.14		<.001; <.001^h^			F (1, 249.4) =11.09		.001; .005^h^			F (1, 243.6) =22.71		<.001; <.001^h^			2.74 (1)		.098; .49^h^
	Time (weeks)		F (1, 216) =11.94		<.001; <.001^h^		F (1, 221) =4.68		.03; .16^h^			F (1, 217.5) =0.49		.49; >.99^h^			F (1, 213.3) =5.82		.02; .08^h^			5.19 (1)		.02; .11^h^
	Treatment × time		F (1, 216) =0.12		.73; >.99^h^		F (1, 221) =0.43		.51; >.99^h^			F (1, 217.5) =3.66		.06; .29^h^			F (1, 213.3) =0.22		.64; >.99^h^			1.39 (1)		.24; >.99^h^

^a^PHQ-9: Patient Health Questionnaire-9.

^b^GAD-7: Generalized Anxiety Disorder 7-Item scale.

^c^WSAS: Work and Social Adjustment Scale.

^d^CD-RISC-10: Connor-Davidson Resilience Scale 10-Item

^e^Baseline scores for PHQ-9, GAD-7, WSAS, and CD-RISC-10.

^f^Receiving treatment for depression at baseline.

^g^Thrive iCBT intervention or control.

^h^*P* values adjusted by the Bonferroni method.

**Table 4:** Effect of the intervention on depression severity, anxiety severity, work/social functioning, and resilience.

Outcome and group			Week 4, LSM^a^ (SE), (95% CI)		Week 8, LSM (SE), (95% CI)		Overall timed-average (weeks 4-8), LSM (SE), (95% CI)		Overall treatment group main effect (weeks 4-8)				
									*F* statistic (*df*)		*P* value		Cohen *d*
**Depression severity** **(PHQ-9^b^)**													
	Intervention group	8.165 (0.373), (7.430 to 8.899)		7.240 (0.385), (6.481 to 7.998)		7.702 (0.336), (7.039 to 8.365)		N/A^c^		N/A		N/A	
	Control group	10.602 (0.371), (9.873 to 11.33)		9.845 (0.367), (9.122 to 10.568)		10.224 (0.328), (9.576 to 10.871)		N/A		N/A		N/A	
	LSM group difference	N/A		N/A		–2.521 (0.471), (–3.448 to –1.593), (–3.743 to –1.298)^d^		F (1, 248)=28.67		<.001; <.001^e^		0.628	
**Anxiety severity** **(GAD-7^f^)**													
	Intervention group	7.121 (0.346), (6.439 to 7.803)		6.481 (0.358), (5.777 to 7.185)		6.801 (0.312), (6.186 to 7.415)		N/A		N/A		N/A	
	Control group	8.724 (0.344), (8.047 to 9.401)		8.382 (0.341), (7.710 to 9.053)		8.553 (0.304), (7.953 to 9.153)		N/A		N/A		N/A	
	LSM group difference	N/A		N/A		–1.752 (0.436), (–2.610 to –0.893), (–2.883 to –0.620)^d^		F (1, 252.8)=16.14		<.001; <.001^e^		0.470	
**Work/social functioning (WSAS^g^)**													
	Intervention group	16.433 (0.602), (15.249 to 17.617)		15.407 (0.621), (14.185 to 16.629)		15.920 (0.542), (14.852 to 16.988)		N/A		N/A		N/A	
	Control group	18.205 (0.597), (17.029 to 19.380)		18.682 (0.592), (17.517 to 19.848)		18.443 (0.529), (17.401 to 19.486)		N/A		N/A		N/A	
	LSM group difference	N/A		N/A		–2.523 (0.757), (–4.016 to –1.031), (–4.487 to –0.558)^d^		F (1, 249.4)=11.09		.001; <.005^e^		0.389	
**Resilience (CD-RISC-10^h^)**													
	Intervention group	24.899 (0.382), (24.148 to 25.652)		25.646 (0.395), (24.868 to 26.424)		25.273 (0.341), (24.601 to 25.945)		N/A		N/A		N/A^c^	
	Control group	22.749 (0.379), (22.003 to 23.496)		23.254 (0.376), (22.514 to 23.995)		23.002 (0.332), (22.346 to 23.657)		N/A		N/A		N/A^c^	
	LSM group difference	N/A		N/A		2.271 (0.476), (1.332 to 3.209), (1.035 to –3.506)^d^		F (1, 243.6)=22.71		<.001; <.001^e^		0.552	

^a^LSM: least squares means.

^b^PHQ-9: Patient Health Questionnaire-9.

^c^N/A: not applicable.

^d^Bonferroni-adjusted 95% CIs.

^e^*P* values adjusted by the Bonferroni method. The adjusted *P* value was associated with the test (F statistic) of the overall timed-average difference of the LSM estimate between the groups (Thrive intervention vs control).

^f^GAD-7: Generalized Anxiety Disorder 7-Item scale.

^g^WSAS: Work and Social Adjustment Scale.

^h^CD-RISC-10: Connor-Davidson Resilience Scale 10-Item.

#### Secondary Outcomes

The results of the main effects (treatment and time) as well as the treatment by time interaction effect from the mixed model and ordinal logistic regression analysis for the secondary outcomes of anxiety, functional impairment, resilience and suicidal ideation are reported in Table 3. Anxiety symptom severity, work and social functional impairment, and resilience were significantly improved for the Thrive intervention group compared to the control group (raw *P*<.001, adjusted *P*<.001 for all; *d*=0.47, *d*=0.39, and *d*=0.55, respectively; Table 4). This pattern was also observed with simple treatment group effects at weeks 4 and 8 (Multimedia Appendix 8).

Suicidal ideation was reported by 41%, 19%, and 16% of participants at baseline, week 4, and week 8, respectively. The predicted odds of increased suicidal ideation (PHQ-9 item 9) for the intervention group showed a lower trend than that of the control group, but did not reach significance (OR 0.55, 95% CI 0.26-1.11, *P*=.10, adjusted *P*=.49; Multimedia Appendix 9). Thrive intervention group participants were 45% and 58% less likely than controls to experience increased suicidal ideation following the entire 8-week follow-up period and at week 8, respectively (Multimedia Appendix 9).

## Discussion

### Principal Results

This study evaluated the effectiveness of a fully automated, stand-alone, video-centric iCBT intervention (Thrive) for reducing depression symptom severity among adults residing in Montana. The design incorporated features of practical, pragmatic, and community-based effectiveness trials: liberal inclusion criteria (mild to severe depression symptoms), allowance for any past and concurrent treatments, and broad (symptom, functional, and resilience) outcomes. Over 8 weeks, depression symptom severity (primary outcome) in the Thrive intervention group was significantly reduced as compared to the control group. Additionally, the Thrive intervention group showed significant improvements in anxiety symptoms, work and social functioning, and resilience compared with the control group.

Although the Thrive intervention, like other iCBT interventions for depression in non-US populations [19], may be equally effective in rural and urban populations, the intervention has several characteristics that may make it appealing to rural US residents as an alternative or supplemental mental health intervention for depression symptoms. A previous qualitative study of Thrive among rural Montana adults led to an adaptation of 6 of the >300 videos to better reflect scenarios and settings common in rural communities [41], which may improve user engagement. Thrive can be performed in the privacy of one’s home, which might offset mental health-related stigma, a frequent barrier to seeking mental health services experienced by rural US residents [51]. Thrive is accessible from any location with internet access and can be used on smartphones, tablets, and computers, making it an easily accessible, cost-effective alternative for rural residents who often have to drive long distances for in-person mental health care [41].

### Comparison With Prior Work

To our knowledge, the clinical effect of the Thrive intervention on depression symptoms is one of the largest reported for an iCBT intervention delivered without clinician interaction. Omitting clinician support allows for broader generalizability to community populations by limiting costs and avoiding barriers associated with an underresourced mental health workforce. A recent meta-analysis of 16 self-guided iCBT studies by Karyotaki et al [21] and a systematic review by Lorenzo-Luaces et al [22] of the same 16 and an additional 5 self-guided iCBT studies reported that 7 studies evaluated iCBT interventions that, like Thrive, had no clinician contact [25-31]. The impact of Thrive on depression symptoms (*d*=0.63) was greater than or similar to the 7 comparable studies (nonsignificant [25]; *d*=0.17 [26]; *d*=0.20 [28]; *d*=0.28 [27]; *d*=0.30 and 0.65 [for analysis of variance and mixed model analyses, respectively] [29]; *g*=0.36 [31]; and *d*=0.50 [30]). The greater impact of Thrive may be a result of the intervention group completing more lessons on average (8.7) than some other fully automated, stand-alone iCBT interventions [24-26].

Compared to self-guided iCBT interventions delivered with meaningful clinician support, described in Karyotaki et al [21] and Lorenzo-Luaces et al [22], the Thrive intervention yielded a similar or greater effect size for depression symptoms: Four studies that used clinician/research personnel contact to determine eligibility found effect sizes of *d*=0.08 [52], *d*=0.38 [53], *d*=0.8 [54], and *g*=0.76 for depression symptoms [55], although the latter study excluded “outliers” from the primary analysis and stated their inclusion resulted in “nonsignificant findings” and reduced the effect size by an undisclosed amount [55]. Two other studies that used iCBT in combination with contact with a therapist also reported similar or lower effect sizes (*d*=0.51 [56] and 0.38 [31]) than those reported for Thrive. Six studies that included individualized email or SMS check-in reminders or telephone diagnostic interviews produced nonsignificant [57] or small-to-medium effect sizes for depression symptoms (*d*=0.34 [30]; *d*=0.36 [58]; *d*=0.55 [59]; *d*=0.57 [60]; and *d*=0.66 [61]). Finally, the six studies providing up to weekly nonautomated email or telephone support reported no significant differences compared to the control for two studies [24,62] and small-to-large significant effects sizes for four studies: (1) *d*=0.22 and *d*=0.34 for support upon request and weekly support, respectively [26]; (2) *d*=0.4 (pre-post within-subjects effect size) [63]; (3) *d*=0.39 [64]; and (4) *d*=1.14 [61].

This study found similar or greater effect sizes for secondary outcomes (anxiety symptoms [*d*=0.47], work and social functioning [*d*=0.39], and resilience [*d*=0.55]) compared to the 21 iCBT trials described in the recent meta-analysis and systematic review [21,22]. In the seven studies reporting anxiety symptom outcomes, four identified nonsignificant differences with the control [53,56,60,62] and three found small-to-nearly moderate effect sizes (*d*=0.22 [54], *d*=0.25 [26], and *d*=0.43 [30]). The three studies that assessed life/work functioning (using the same measure reported here [WSAS]) found a nonsignificant difference as compared to the control [62], a significant improvement for iCBT+treatment as usual but not in iCBT compared to the control (effect sizes not provided) [52], and an effect size of 0.36 [29]. Two other studies found no significant difference [57] and an effect size of 0.65 [54] when measuring life/work functioning with the Sheehan Disability Scale. To our knowledge, no other iCBT studies have evaluated resilience. Lastly, this study found a nonsignificant reduction (58%) in the odds of having greater suicidal ideation in the Thrive intervention group than in the control group at 8 weeks. A small effect size (*d*=0.20) was found in the single prior study that measured the impact of an iCBT intervention on suicidality [58].

The design of this study supports the generalizability of its findings in community settings. First, the minimal eligibility restrictions indicate the potential applicability of the Thrive intervention in general populations outside the controlled research setting. Of the seven studies described in Karyotaki et al [21] and Lorenzo-Luaces et al [22], only one that delivered a fully automated, stand-alone iCBT intervention like the Thrive intervention had similarly broad eligibility criteria [29]. Second, omitting clinician-administered diagnostic evaluations, as used in several iCBT studies [57,59-61], allows for broader generalizability to community settings. The use of person-administered diagnostics greatly increases costs, limits broad intervention dissemination due to an insufficient mental health workforce, and decreases the potential public health impact of fully automated, stand-alone iCBT interventions.

### Limitations

This study had several limitations. Like most iCBT studies [21], assessment completion rates were low, with 68% and 65% of randomized subjects completing assessments at 4 and 8 weeks, respectively. These rates are slightly below the 73% (range 55%-95%) unweighted mean completion rate [24-30,52,55,58-64] for short-term (6-16 weeks) follow-up assessments of all studies in Karyotaki et al [21]. Additionally, the study offered monetary incentives for 4- and 8-week survey completion, which are associated with greater response in electronic questionnaires [65] and retention rates [66], which limits conclusions about uptake of Thrive in nonresearch settings. Monetary incentives have been used in all six RCTs of iCBT interventions for depression in US populations [24,25,27,28,54,57] and three of the seven (43%) RCTs of fully automated, stand-alone iCBT interventions reported in Karyotaki et al and Lorenzo-Luaces et al [21,22,25,27,28]. Although using self-assessments, a common practice in iCBT studies, is a potential weakness of the study, the use of validated, widely used instruments largely addressed this issue. The PHQ-9 (depression symptoms), Generalized Anxiety Disorders Scale-7 (anxiety symptoms), and WSAS-5 (functioning) correlate well with clinician-administered instruments [37,67,68] and are sensitive to interventional effects [45,69,70]. Of note, self-assessments may underestimate the effect of iCBT relative to similar studies using clinician-administered assessments [71]. The study eligibility, which omitted the need for a depressive disorder diagnosis and included participants across the full range of depression symptom severity increases the study’s generalizability. However, the make-up of the recruited study population, with high a proportion of female (85%) and white (93%) participants with at least some education after high school (69%) limits its generalizability. Greater participation of women is a common limitation among studies of iCBT for depression. For example, Karyotaki et al [21] reported that 66% of all participants were female, and among the studies of fully automated, stand-alone iCBT interventions, female participation ranged from 65% to 81% [21]. The high proportion of female participants across iCBT studies could be due, in part, to the greater prevalence of depression in women [2] or as a yet-unidentified barrier for men. The relatively high percentage (23.5%) of fraudulent participants identified is another limitation, which is becoming increasingly common in internet-based studies [72]. The number of fraudulent participants in this study is similar to that in several other studies using Web-based surveys: 28.7% [73], 20.5% [74], and 18.7% [75]. Although the study was powered appropriately to detect between-group differences in a priori defined primary and secondary outcomes, even when allowing for corrections for multiple testing (design strength of this study), it was underpowered to detect meaningful difference in the post-hoc assessment of suicidal ideation, as only 41% of subjects experienced suicidality at baseline. In addition, the 8-week evaluation period cannot inform on the long-term impact of the Thrive intervention when delivered in community settings. Longitudinal studies with longer follow-up periods are warranted to determine whether iCBT programs impact the risk of depression relapse [10]. Finally, as a community-based trial, these findings cannot be generalized to clinical settings.

### Conclusions

Thrive, a fully automated, stand-alone iCBT intervention, demonstrated greater short-term improvements in depression and anxiety symptoms, work and social functioning, and resilience compared to control participants in a broad community population with mild to severe depression symptoms. A trend toward greater reduction in suicidal ideation was also observed. The magnitude of clinical benefit seen with the Thrive intervention appears to be greater than most other fully automated, stand-alone interventions and similar to those requiring clinician support. Thus, Thrive represents a potentially effective and cost-effective solution for treating depression symptoms in rural regions that often have few or no mental health providers. Evaluations of the impact of the Thrive intervention over longer periods of time and other population settings are warranted.

## Appendix

Multimedia Appendix 1 Informed consent.

Multimedia Appendix 2 Description of study population with definition and removal of fraudulent participants.

Multimedia Appendix 3 CONSORT diagram.

Multimedia Appendix 4 Study safety protocol.

Multimedia Appendix 5 Covariates.

Multimedia Appendix 6 Power calculations.

Multimedia Appendix 7 Baseline PHQ-9 scores.

Multimedia Appendix 8 Adjusted least mean squares for secondary outcomes.

Multimedia Appendix 9 Effect of the Thrive intervention on suicidal ideation.

Multimedia Appendix 10 Participant flow chart.

Multimedia Appendix 11 CONSORT‐EHEALTH checklist (V 1.6.1).

## Abbreviations

CDRISC-10: Connor Davidson Resilience Scale-10
DSM: Diagnostic and Statistical Manual of Mental Disorders
GAD-7: Generalized Anxiety Disorder 7-item scale
iCBT: internet-based cognitive behavior therapy
LSM: least squares means
OR: odds ratio
PHQ-9: Patient Health Questionnaire-9
RCT: randomized controlled trial
WSAS-5: Work and Social Adjustment Scale-5
