# Supplementary material for: Use of a Fully Automated Internet-Based Cognitive Behavior Therapy Intervention in a Community Population of Adults With Depression Symptoms: Randomized Controlled Trial
Source: J Med Internet Res. 2019 Nov 18;21(11):e14754. doi: 10.2196/14754 (PMC6887812; doi:10.2196/14754)
Supplement: Multimedia Appendix 1 [file jmir_v21i11e14754_app1.docx]

## **Multimedia Appendix 1. Informed Consent**

[Page Title] Study Application (page 2 of 2)

Thank you. You are eligible to participate in the Thrive for Montana study. Before deciding whether to participate, please read this “informed consent” form, which tells you about the purpose, risks, and benefits of this research study. If you decide to participate, follow the instructions at the bottom of the page to complete the form.

**SUBJECT ELECTRONIC CONSENT FORM FOR PARTICIPATION IN HUMAN RESEARCH AT MONTANA STATE UNIVERSITY**

**Project Title**

Randomized Controlled Trial of a culturally-adapted version of Thrive, a computerized Cognitive Behavior Therapy (cCBT) program to treat depressive symptoms, syndromes, and disorders among rural Montanans

**Funding Source**

Montana INBRE (IDeA Network for Biomedical Research Excellence) and National Institutes of Health Award# P20GM103474

**Principal Investigator:**

Mark Schure, Ph.D.

Center for Mental Health Research & Recovery

Montana State University

305 Herrick Hall

Bozeman, MT 59717

**Invitation**

You are invited to take part in this research study. The information in this form is meant to help you decide whether or not to take part. If you have any questions, please ask.

**Why are you being asked to take part in this study?**

You are being asked to be in this study because your self-assessment identified depressive symptoms and you met all enrollment requirements. This study will help determine the effectiveness of Thrive, an interactive computerized Cognitive Behavior Therapy (cCBT) program, to help treat depressive symptoms, syndromes, and disorders.  Both therapist-guided and computerized Cognitive Behavior Therapy have been shown to be effective in reducing depressive symptoms for people with wide levels of depression severity.

**What is the reason for doing this research?**

Thrive is an advanced cCBT program using largely video format to explain and convey messages that aim to help users improve their lives.  The purpose of this study is to determine how well the Thrive program reduces depressive symptoms.

**What will I be expected to do during this research study?**

This study is designed so that participants can easily, efficiently, and confidentially use the Thrive program and complete periodic brief self-assessments online. If you decide to take part in this study, we will ask you to provide your electronic signature at the end of this consent form.  Once you have read and agree to requirements of this study, you will be expected to do the following:

1. Provide your first and last name, your zip code, general demographic information, and an email address where you wish to receive personalized access to the Thrive program.

1. You will be randomly assigned to the group receiving immediate access to the Thrive program OR assigned to the group receiving access to Thrive program in 8 weeks.  This random assignment permits us to compare the difference in depression symptoms after 8 weeks of Thrive use.  For those with delayed access to Thrive, we will provide access to information about depression. Whether immediately or in 8 weeks, you will receive an email with an invitation to enroll. You will be expected to spend time interacting with the program to which you are randomly assigned throughout the next 8-weeks.

1. Regardless of your randomized assignment to immediate or delayed enrollment with access to Thrive, you will be asked to complete brief confidential online self-assessments immediately upon enrolling in the study, with follow-up assessments at 4 and 8 weeks after enrollment.  Delayed enrollment participants will be asked to complete another two assessments at 12 and 16 weeks. All participants will be asked to complete additional final assessments 4 and 10 months after the 8-week trial period of Thrive.  For each **completed** assessment you will be compensated by an Amazon email code worth $25.  For completing the assessments at the 8^th^ week of using Thrive*,* the value of the Amazon email code is $30.

**What are the possible risks of being in this research study?**

You may feel uncomfortable thinking about your depression symptoms and negative thoughts associated with depression, or may experience anxiety when carrying out cognitive behavior therapy tasks. The program may not be effective for your depressive symptoms; however, if you decide you need additional assistance managing your depressive symptoms or have suicidal thoughts, we suggest that you:

- Call your doctor
- Visit an emergency room
- Call 911
- Call the National Suicide Prevention Lifeline at 1-800-273-TALK (8255)
- Text “Matters” to 741741 to start a text chat with the Crisis Text Line, or
- Visit NowMattersNow.org to learn skills for dealing with thoughts of suicide
- Visit the Montana National Alliance for Mental Illness website at namimt.org/county-resource-guides

**Can I continue to see my mental health provider and take my medications?**

Yes, we encourage you to use any care available in helping with your depression symptoms.  Throughout the study you may continue to receive treatment with any mental health provider and continue taking prescribed medications.

**What are the possible benefits to you of being in this research study?** 
The treatment may be effective for you and as a result, your depressive symptoms may improve.

**What are the possible benefits to other people?** 
If the Thrive program is good at helping people with self-help treatment for depressive symptoms, it may help other patients in the future. If successful, the program should enable wider availability of this treatment, which will enable greater numbers of people with depressive symptoms to be treated, which in turn may improve long-term outcomes and quality of life. Greater availability of Thrive, if it is effective, may be especially helpful where therapists are less available. 
 
**What are the alternatives to being in this research study?**

 Your participation in this study is entirely voluntary.  Effective treatments for depression symptoms include medication and psychotherapy and may be available. You are free to decline participation in this study now or at any time during the study should you decide to stop participating. 
 
**What will participation in this research study cost you?** 
There is no cost to you to be in this research study.

**Will you be compensated for being in this research study?** 
Yes. After completing each follow-up assessment, you will be compensated immediately with an Amazon voucher sent to your email with a link to an Amazon Gift Code. All follow-up assessment completion vouchers will be for $25 except the last one which will be for $30.

**How will your information be protected?**

To ensure confidentiality of records collected from this study, your personal information will be automatically separated from your self-assessment data.  Electronic data will not contain any individual identifiers, and will be password protected.  Data will only be accessible to the Principal Investigator and Study Coordinator.  All identifying information will be kept secure on password protected computers/drives and safe-locked cabinets. Group data on mean scores will be coded, but not traceable to specific individuals. 

**Computer Interview security** 
Security and privacy have been of the utmost concern in the development of the computer interview. No personally identifiable user information is held in the application or database. The application will require the combination of a username and password in order to access the material. Data security within the server database is ensured through two levels of authentication. The application will also be hosted on a Web server maintained by a firm that specializes in Web hosting, ensuring that all software and hardware security is current.

If you have questions about this research project, you can contact Mark Schure at (406) 994-3248 (mark.schure@montana.edu). If you have additional questions about your rights of being involved in this project, you can contact the Chair of the Institutional Review Board, Mark Quinn, (406) 994-4707 (mquinn@montana.edu).

Disclosure: Co-Investigator Dr. John Greist previously held a financial interest in Waypoint Health Innovations, which developed the Thrive intervention that this research project is evaluating. He no longer has a direct financial interest in Waypoint Health Innovations, but does retain a small interest in Waypoint Health Innovations through Healthcare Technology Systems where Dr. Greist is CEO and a shareholder. He is also a consultant to Waypoint on projects outside of the Montana State University grant. The terms of Dr. Greist’s financial relationship with Waypoint Health Innovations have been reviewed by Montana State University, and his involvement with this research project has been approved in accordance with its conflict of interest policies.

**Consent:**  
I have read the information in this consent form. I have reviewed any questions with the project staff and have had them answered to my satisfaction. I voluntarily agree to participate.  
 
**Documentation of informed consent**
You are freely making a decision whether to be in this research study. Clicking "Yes" to answer the question on the next page means that (1) you have read and understood this consent form, (2) any additional questions have been answered, and (3) you have decided to be in the research study. 

**Please print this page for your records.**

Entering the information below and clicking “Join Study” represents your legal signature and will complete your enrollment in the Thrive for Montana Study.

Your first name ______________________

Your last name ______________________

Your email address ______________________

Your phone number ______________________

Your date of birth: [show month, day, year from screening page]

Your ZIP code: [show zip code from screening page]

[Button] Print

[Button] Join Study

[When the prospective participant clicks “join study” we will validate that every field has been completed and that the phone number and email fields have the correct format (e.g. name@domain.com for the email field). If all fields are complete, we will store the entire page – the informed consent text and the user’s input – in a record, timestamp it, and encrypt it. Researchers would have access to the completed IC forms to print out for their records and/or mail to patients who wanted a copy.]

[If Informed Consent is successfully completed, the Assessment Website will assign a Study ID to user and randomly assign user to Treatment or Control group.]

[Treatment Group: If participant is assigned to Treatment Group, in the background the Assessment Website will immediately pass the participant’s name, Study ID, and email address to Thrive, and Thrive will create an account for that person using his/her Study ID as his/her Thrive Enrollment Token and automatically send an email invitation to him/her.]
