# Supplementary material for: Use of a Fully Automated Internet-Based Cognitive Behavior Therapy Intervention in a Community Population of Adults With Depression Symptoms: Randomized Controlled Trial
Source: J Med Internet Res. 2019 Nov 18;21(11):e14754. doi: 10.2196/14754 (PMC6887812; doi:10.2196/14754)
Supplement: Multimedia Appendix 2 [file jmir_v21i11e14754_app2.docx]

**Multimedia Appendix 2.** **Description of study population with definition and removal of fraudulent participants**

Four hundred sixty-three individuals met the study eligibility criteria and were enrolled (see Multimedia Appendix A3). Fraudulent participants are becoming increasingly common with internet-based studies using self-reported measures and material incentives, and they can lead to invalid or unreliable data [1]. To identify fraudulent accounts within the current study, the authors reviewed all participants’ intervention accounts using the criteria described below that was determined by a review panel (first author, 1 research assistant, and 1 faculty member at Montana State University). Many of the methods used to identify fraudulent cases (“fraudsters”) were mentioned in Teitcher *et al.* [1] and used in a recent study by Ballard *et al.* [2], and all methods were approved by the Montana State University Institutional Review Board prior to use.

In total, 109 of the 463 (23.5%) enrolled participants were determined to be fraudulent. The percentage of “fraudsters” identified in the current study is similar to a recent clinical study using web-based surveys (28.7%) [2]. Of the 109 “fraudsters”, 24 (22%) used non-USA IP (internet protocol) addresses; 11 (10%) used IP addresses strongly suspected of being inauthentic using data from GetIPIntel.net; 18 (17%) provided invalid or out-of-state business phone numbers and 2 (2%) provided identical names, phone numbers, ZIP Codes, and dates of birth.

The remaining 54 fraudulent participants were identified using multiple criteria as described in the table below with the number of fraudulent participants in each category listed.

| Fake reason A (n=32) | Montana business phone numbers | |
| --- | --- | --- |
|  | Similarities to other participants with England, Australia, Canada, and Ireland IP addresses: | |
|  | - Identical rare (0.5% market share) browser version | |
|  | - Names don't resemble email addresses | |
|  | - Identical answers to gender, race, marital status, employment, education, and insurance demographics questions^a^ | |
|  | - Very similar Thrive usage patterns | |
|  | - Enrolled in study during the same 4-day window | |
| Fake reason B (n=4) | Montana business phone numbers | |
|  | Similarities to other participants with Canada or inauthentic IP addresses: | |
|  | - Identical rare (0.02% market share) browser version | |
|  | - Identical answers to gender, marital, employment, education, and insurance demographics questions^a^ | |
|  | - Enrolled in study during same 4-day window | |
| Fake reason C (n=2) | Similarities to other participants with China IP addresses: | |
|  | - Identical rare (0.16% market share) browser version - Phone numbers don't match possible identity | |
|  | - Click-shy^b^ | |
|  | - Enrolled in study immediately after a fraudulent participant completed baseline assessments | |
| Fake reason D (n=2) | Montana business phone number | |
|  | Similarities to other participant with inauthentic IP address: | |
|  | - Identical rare (0.03% market share) browser version | |
|  | - Identical answers to gender, marital, employment, education, and insurance demographic questions^a^ - Enrolled in study during same 6-day window | |
| Fake reason E (n=9) | Similarities to other participants with China IP addresses:   - Non-USA or non-Montana phone number | |
|  | - Click-shy^b^ | |
|  | - Whitepages rates email address "untrusted" - Email address did not resemble the participant’s name | |
|  | - Majority of activity between 1am and 6am | |
| Fake reason F (n=1) | MT business phone number | |
|  | Similarities to other participant with inauthentic IP address   - Identical rare (0.02% market share) browser version | |
|  | - Identical answers to gender, race, marital, employment, education, and insurance demographics questions^a^ | |
|  | - Enrolled in study during same 24-hour period | |
| Fake reason G (n=1) | Whitepages rates email address "untrusted" | |
|  | Whitepages has no record of phone number | |
|  | Whitepages has no record of someone with that name and age in US | |
|  | Click-shy^b^ | |
|  | Email address did not resemble the participant’s name | |
|  | Majority of activity between 1am and 6am | |
| Fake reason H (n=1) | Whitepages rates email address as "untrusted" | |
|  | Whitepages says phone is registered to government organization | |
|  | Whitepages has no record of someone with that name and age in US | |
|  | Click-shy^b^ | |
|  | Email address did not resemble the participant’s name | |
|  | Majority of activity between 1am and 6am | |
| Fake reason I (n=2) | | Same email domain for college outside Montana, but ages are 38 and 48 |
|  | | Area code indicates same state (not Montana)  Whitepages rates email address "untrusted" |
|  | | Email addresses did not resemble the participants’ names |
|  | | Click-shy^a^ |
|  | Majority of activity between 1am and 6am | |

^a^ Demographic profiles were identical to some used by individuals with foreign or inauthentic IP addresses.

^b^ Accessed the study website without clicking on links in emails sent to participants, a technique that helps obfuscate the IP address

**Additional Exclusions of Study Participants from Analyses**

Three individuals provided invalid email addresses excluding them from study participation, and data from 2 participants in the control group were discarded as they were accidentally provided immediate access to the intervention. Six participants were excluded because of missing baseline data on the covariate “currently receiving psychosocial therapy for depression,” which was required for data analysis.

1. Teitcher JE, Bockting WO, Bauermeister JA, Hoefer CJ, Miner MH, Klitzman RL. Detecting, preventing, and responding to "fraudsters" in internet research: ethics and tradeoffs. J Law Med Ethics. 2015 Spring;43(1):116-33. PMID: 25846043. doi: 10.1111/jlme.12200.
2. Ballard AM, Cardwell T, Young AM. Fraud Detection Protocol for Web-Based Research Among Men Who Have Sex With Men: Development and Descriptive Evaluation. JMIR Public Health Surveill. 2019 Feb 4;5(1):e12344. PMID: 30714944. doi: 10.2196/12344.
