# Supplementary material for: Use of a Fully Automated Internet-Based Cognitive Behavior Therapy Intervention in a Community Population of Adults With Depression Symptoms: Randomized Controlled Trial
Source: J Med Internet Res. 2019 Nov 18;21(11):e14754. doi: 10.2196/14754 (PMC6887812; doi:10.2196/14754)
Supplement: Multimedia Appendix 3 [file jmir_v21i11e14754_app3.docx]

**Multimedia Appendix A3**. **CONSORT diagram**

## Enrollment

Randomized (n= 463)

**Excluded (n= 110)**

- Inclusion criteria not met (n= 47)
- Declined to participate (n= 63)

Assessed for eligibility (n=573)

Analyzed (n= 181)

- Excluded from analysis (n=66)
- Fake participant (n=61)
- Incomplete BL survey (n=4)
- Invalid email (n=1)

Week 4 Week 8

Lost to Follow-Up 65 76

- PHQ9 survey not completed

**Allocated to intervention group (n=247)**

- Received allocated intervention (**n=181**)
- Missing data or excluded (**n=66**)
- Incomplete BL survey (n=4)
- Fake participant (n=61)
- Invalid email address (n=1)

## Analysis

## Allocation

## Follow-Up

Analyzed (n=162)

- Excluded from analysis (n=54)
  - Fake participant (n=48)
  - Incomplete BL survey (n=2)
  - Program error (n=2)
  - Invalid email (n=2)

Week 4 Week 8

Lost to Follow-Up 46 43

- PHQ9 survey not completed

**Allocated to control group (n=216)**

- Completed baseline PHQ9 (**n=162**)
- Missing data or excluded (**n=54**)
- Incomplete BL survey (n=2)
- Fake participant (n=48)
- Program error (n=2)
- Invalid email address (n=2)

♦ Did not receive allocated intervention (invalid email, n=2)
