# Supplementary material for: Use of a Fully Automated Internet-Based Cognitive Behavior Therapy Intervention in a Community Population of Adults With Depression Symptoms: Randomized Controlled Trial
Source: J Med Internet Res. 2019 Nov 18;21(11):e14754. doi: 10.2196/14754 (PMC6887812; doi:10.2196/14754)
Supplement: Multimedia Appendix 5 [file jmir_v21i11e14754_app5.docx]

**Multimedia Appendix 5. Covariate analysis**

An initial pool of 10 characteristic variables was selected *a priori* for analysis as potential covariates of depression severity, anxiety, resilience, functional impairment, and suicidal ideation. The pool of potential covariates included age (years), educational attainment (≤high school/trade school, college up to Bachelor’s degree, ≥Master’s degree), employment status (employed vs. not employed), marital status (married vs. not married), health insurance (private vs. public), rural status (RUCA classification of urban, large rural, small rural, isolated), and receiving other treatment (e.g., receiving therapy for depression, receiving therapy for anxiety, taking medication for depression, taking medication for anxiety; response options of ‘yes’/ ‘no’). Race, sex, and veteran status were not included in the potential covariate pool, because the study sample was predominately white (93%), female (85%), non-veteran (95%).

Starting with the initial pool of 10 variables, along with respective baseline outcome measure (PHQ, GAD, WSAS, CD-RISC), a filtering process was used to identify a subset of covariates that seemed to contain predictive power for each outcome. The process was implemented using the adaptive Lasso-penalized variable selection method [1], with the Schwarz Bayesian information criterion, in the context of a linear regression model for the outcomes of depression severity, anxiety, functional impairment, resilience, and suicidal ideation that was based on 10,000 bootstrap samples. The goal of the adaptive LASSO-penalized linear regression was to select a parsimonious and well-fitting subset of potential predictors (covariates) of each outcome by performing simultaneous variable selection and parameter estimation. The “best” subset selection of regressors (covariates) for each outcome that emerged from this supervised variable selection (filtering) process included: Respective baseline outcome measure (PHQ, GAD, WSAS, CD-RISC) and receiving therapy for depression (yes/no). Of note, the 95% bootstrap confidence interval for each of these two covariates did not contain zero (at α = 0.05). These two variables were included as covariates in the statistical models for the primary and secondary outcomes.

1. Zou H. The adaptive lasso and its oracle properties. Journal of the American Statistical Association. 2006;101(476):1418-29.
