# Supplementary material for: Use of a Fully Automated Internet-Based Cognitive Behavior Therapy Intervention in a Community Population of Adults With Depression Symptoms: Randomized Controlled Trial
Source: J Med Internet Res. 2019 Nov 18;21(11):e14754. doi: 10.2196/14754 (PMC6887812; doi:10.2196/14754)
Supplement: Multimedia Appendix 6 [file jmir_v21i11e14754_app6.docx]

**Multimedia Appendix 6. Power calculations**

The *a priori* evaluable sample size proposed was 234 participants (117 per group) for the repeated measures design/analysis in the current study. The sample size calculation anticipated a dropout rate of ~49% within the first 4 weeks (period designating an evaluable subject) after enrollment, based on prior, similar studies of the Thrive intervention. Thus, 459 participants were to be enrolled to allow for the expected rate of attrition with the intent of capturing a range of evaluable data for 234 participants. Utilizing linear mixed models, contrasts of means, tests of regression coefficients, and a total of 234 participants, we estimated statistical power of 80% to detect an effect size as small as *d*=0.30 attributable to any single treatment group mean contrast, based on an overall alpha level of 0.05.
