# Supplementary material for: Use of a Fully Automated Internet-Based Cognitive Behavior Therapy Intervention in a Community Population of Adults With Depression Symptoms: Randomized Controlled Trial
Source: J Med Internet Res. 2019 Nov 18;21(11):e14754. doi: 10.2196/14754 (PMC6887812; doi:10.2196/14754)
Supplement: Multimedia Appendix 7 [file jmir_v21i11e14754_app7.docx]

**Multimedia Appendix 7.** **Baseline depression symptom severity**

| Depression Symptom Severity^a^ | Overall  N=343 | | Intervention  N=181 | | Control  N=162 | |
| --- | --- | --- | --- | --- | --- | --- |
|  | N | % | N | % | N | % |
| Mild | 88 | 25.7 | 46 | 25.4 | 42 | 25.9 |
| Moderate | 109 | 31.8 | 55 | 30.4 | 54 | 33.3 |
| Moderately severe | 99 | 28.9 | 54 | 29.8 | 45 | 27.8 |
| Severe | 47 | 13.7 | 26 | 14.4 | 21 | 13.0 |

^a^ PHQ-9 score ranges: Mild = 5-9; Moderate = 10-14; Moderately severe = 15-19; Severe = 20-27.
