# Supplementary material for: Use of a Fully Automated Internet-Based Cognitive Behavior Therapy Intervention in a Community Population of Adults With Depression Symptoms: Randomized Controlled Trial
Source: J Med Internet Res. 2019 Nov 18;21(11):e14754. doi: 10.2196/14754 (PMC6887812; doi:10.2196/14754)
Supplement: Multimedia Appendix 9 [file jmir_v21i11e14754_app9.docx]

**Multimedia Appendix 9. Effect of Thrive intervention on suicidal ideation.**

| Outcome and Group^a^ | Odds of Suicidal Ideation at Week 4 | | | | | Odds of Suicidal Ideation at Week 8 | | | | Overall Odds of Suicidal Ideation | | | |
| --- | --- | --- | --- | --- | --- | --- | --- | --- | --- | --- | --- | --- | --- |
|  | | OR^b^ | 95% CI | Z statistic | *P* value | OR^b^ | 95% CI | *Z* statistic | *P* value | OR^b^ | 95% CI | Z statistic | *P* value |
|  | |  |  |  |  |  |  |  |  |  |  |  |  |
| Treatment Group | | 0.719 | 0.326, 1.585 | 0.820 | 0.41^c^ | 0.416 | 0.168, 1.025 | 1.900 | 0.057^d^ | 0.547 | 0.267, 1.112^e^ | 1.650 | 0.098^f^ |

^a^ The change over time in Suicidal Ideation (Item 9 on PHQ-9) was compared between the two treatment groups (Thrive intervention vs. Control) using an ordinal logistic regression model via a Generalized Estimating Equation analysis of repeated measures, while adjusting for the baseline measure of suicidal ideation (PHQ-9 item 9) and receiving therapy for depression at baseline (yes/no). The cumulative probabilities were modeled over the higher-ordered suicidal ideation scale score (more suicidal ideation). An estimated Odds Ratio (OR) < 1 indicated lower predicted odds of suicidal ideation for Thrive intervention vs. Control.

^b^ Odds Ratio (OR)

^c^ Bonferroni Adjusted *P* = 1.00

^d^ Bonferroni Adjusted *P* = 0.28

^e^ Bonferroni-adjusted 95% confidence interval = 0.212 to 1.40

^f^ Bonferroni Adjusted *P* =0.49
