# Supplementary material for: Use of a Fully Automated Internet-Based Cognitive Behavior Therapy Intervention in a Community Population of Adults With Depression Symptoms: Randomized Controlled Trial
Source: J Med Internet Res. 2019 Nov 18;21(11):e14754. doi: 10.2196/14754 (PMC6887812; doi:10.2196/14754)
Supplement: Multimedia Appendix 10 [file jmir_v21i11e14754_app10.docx]

**Multimedia Appendix 10. Participant Flowcharts**

Home Page 001

Informed Consent 008

GAD7 (baseline) 009

Next Steps – Treatment (baseline) 014

Next Steps– Control (baseline) 013

Link to Thrive enroll page

Study group?

0

1-3

Control

Treatment

WSAS (baseline) 010

PHQ9 9^th^ item?

Can you stay safe? (screening) 005

Stay safe?

Please get help immediately (ineligible) 007

No

How to get help (eligible) 006

Yes

*Assign study ID and Randomize. If treatment: create Thrive account.*

**New Participant flow**

Demographics (baseline) 012

CD-RISC10 (baseline) 011

Study application (screening) 002

Link to NIMH

Not MT or age<18 or no tech or PHQ9 0-4

Eligible?

MT and age>=18 and has tech and PHQ9 5-27

Thank you for applying (ineligible) 003

PHQ9 9^th^ item?

How to get help (ineligible) 004

0

1-3

Home Page 001

Valid last name and birth month

PHQ9 (returning participant) 103

PHQ9 ninth item?

0

1-3

Not inside user’s Assessment Window

GAD7 (returning participant) 109

**Returning Participant flow**

Here, tenure means days since enrolling in study.

How to get help (returning participant) 104

WSAS (returning participant) 110

CD-RISC10 (returning participant) 111

Study group and tenure

Next Steps – Treatment (4 weeks) 113

Study tenure?

Inside user’s Assessment Window

No survey today 102

Next Steps – Treatment (8 weeks) 114

Next Steps – Treatment (26 weeks) 115

Finished – Treatment (52 weeks) 116

Next Steps – Control (4 weeks) 117

Next Steps – Control (8 weeks) 118

Next Steps – Control (12 weeks) 119

Next Steps – Control (16 weeks) 120

Next Steps – Control (34 weeks) 121

Finished – Control (60 weeks) 122

Treatment, <=55 days

Treatment, 56-181 days

Treatment, 182-363 days

Treatment, >=364 days

Control, <=55 days

Control, 56-83 days

Control, 84-111 days

Control, 112-237 days

Control, 238-419 days

Control, >=420 days

Demographics (returning participant) 112
